# Cardiac Sarcoidosis - Arrhythmias, Inflammation and Anti-inflammatory Drug Therapy

**DOI:** 10.1016/s0972-6292(16)30562-9

**Published:** 2012-12-02

**Authors:** Kartikeya Bhargava

**Affiliations:** Senior Consultant Cardiology, Medanta-The Medicity, Gurgaon, Delhi- NCR, Haryana, India

**Keywords:** Cardiac Sarcoidosis, Arrhythmias

Sarcoidosis is a multi-system granulomatous disease of unclear etiology with variable presentation. The common sites of involvement include the lungs and the lymphnodes, though many other organs including liver, spleen, skin, eyes, and even the heart can get involved. The pathological hallmark of this disease is a non-caseating granuloma.

The prevalence of cardiac involvement in patients with systemic sarcoidosis ranges from 3.7 to 54.9% depending on the population studied (asymptomatic or symptomatic), imaging techniques used and criteria used for diagnosis.[1] Though cardiac involvement may be asymptomatic, common cardiac manifestations include conduction abnormalities usually complete atrioventricular block, ventricular arrhythmias, heart failure, atrial tachyarrhythmias and sudden cardiac death. Rarely, isolated cardiac sarcoidosis without manifestations related to other organs occurs and presents difficulties in the diagnosis. Since the yield of endomyocardial biopsy for cardiac sarcoidosis is low, due to patchy and focal involvement of myocardium, the diagnosis of cardiac sarcoidosis is often made by presence of cardiac manifestations along with tissue diagnosis from other organs. No international consensus guidelines or diagnostic criteria for cardiac sarcoidosis exist except for the Japanese Ministry of Health and Welfare Criteria.[2,3] To compound the difficulties with the diagnosis, tuberculosis affecting the heart can have very similar clinical [ventricular tachycardia (VT), lymphnode enlargement], radiological (mediastinal adenopathy, lung lesions) and imaging features [(mid myocardial scar, delayed enhancement on magnetic resonance imaging (MRI) or focal uptake of 18flouro-deoxyglucose (FDG) on positron emission computerized tomography (PET CT)]. The histology showing caseating granulomas (in lung or node biopsy) or positive stain or culture for acid-fast bacilli and/or positive DNA-PCR for Mycobacterium tuberculosis in the tissue can indicate the correct diagnosis of tuberculosis and guide appropriate disease specific therapy.[4] The differentiation is important since steroid administration without anti-tubercular therapy can result in flaring of the tuberculosis if underlying tuberculosis was present. A high index of suspicion is essential for the diagnosis of both tuberculosis and sarcoidosis of the heart. There is also a possibility of co-existence of the two diseases (tuberculosis-sarcoidosis overlap), especially in developing countries like India with high prevalence of tuberculosis.

 Even when the diagnosis is confirmed, the treatment of cardiac sarcoidosis poses a lot of challenges to the clinician. Though, atrioventricular block, the most common manifestation of cardiac sarcoidosis, may reverse with steroid and/or immunosuppressive therapy, the recurrence rate is very high and pacemaker implantation is almost always needed.[5] The management of VT is even more complex as has been shown in the case report published by Ajay M Naik et al.[6] in this issue of the journal. The VTs in cardiac sarcoidosis are usually monomorphic and rarely polymorphic. However, spontaneous or inducible monomorphic VTs of multiple QRS morphologies indicating different exit sites or multiple reentrant circuits due to patchy myocardial involvement are often present. The pathogenesis of these VTs relates to either acute granulomatous inflammation in the myocardium seen in the early active phase of sarcoidosis or late myocardial scarring in the inactive phase of the disease. Interestingly, both healed granulomas and varying degrees of active inflammation may co-exist producing a heterogenous substrate that is unique to this disease.[7] The disease specific therapy including corticosteroids and steroid sparing immunosuppressive agents (like methotrexate, azathioprine, cyclophosphamide, infliximab, anti-malarials, and thalidomide) is likely to reduce the VT occurrence during the active inflammatory phase but unlikely to help in the late scar related reentrant VTs. The reports of radiofrequency (RF) ablation for VT in cardiac sarcoidosis have been mixed - with some reporting good results and others reporting poor efficacy.[8,9] Also, for it to be successful, both endocardial and epicardial approaches to ablation may be required in some cases.[8] It is logical that inflammation related VT during the active phase of sarcoid is likely to have poor result of RF ablation in absence of anti-inflammatory drug therapy, whereas scar related VT is likely to respond better to RF ablation. Knowing whether sarcoid is currently in active or scar phase is itself challenging. Increased uptake on FDG PET suggests acute inflammation where as delayed enhancement on MRI indicates a scar. Role of high-sensitivity C reactive protein (hs-CRP), erythrocyte sedimentation rate (ESR) and angiotensin converting enzyme (ACE) levels to assess activity is not well defined but may be useful in individual cases. Ajit Thachil et al. have proposed a working strategy for diagnosis and management of monomorphic VT due to granulomatous heart disease (sarcoidosis and tuberculosis).[4]

Atrial arrhythmias are uncommonly seen in cardiac sarcoidosis.[10] Here again, the pathogenesis may involve both inflammation as well as scarring in the left and right atrium. The report of two cases of atrial flutter in patients with cardiac sarcoidosis by Namboodiri et al.[11] where in steroid therapy rendered the flutter non-inducible in the first case and facilitated the successful ablation in the second case with extensive left atrial scarring, again suggests a major role of inflammation in origin of arrhythmias in cardiac sarcoidosis. Similarly, the case report of Uma Srivatsa et al.[12] where cardiac sarcoidosis presented with atrial fibrillation (AF) and later progressed to other manifestations like AV block, myocardial involvement and cardiomyopathy also highlighted the interlink with inflammation and reduction of AF burden with anti-inflammatory disease specific therapy. The case series and reports of these nature and clinical registries are very important for understanding the clinical patterns, result of investigations and guiding therapy in disorders like sarcoidosis where no large scale clinical studies exist and are not likely to be possible in future due to logistic reasons.

With increasing recognition of role of inflammation in cardiac arrhythmias, cardiac sarcoidosis provides an important interlink. As our understanding of inflammatory diseases affecting the heart like tuberculosis and sarcoidosis grows, more research on the inflammatory markers to guide therapy and on agents targeting inflammation for treatment of cardiac arrhythmias in these diseases is the need of the hour.
